# Limits to the Rational Production of Discourse Connectives

**DOI:** 10.3389/fpsyg.2021.660730

**Published:** 2021-05-28

**Authors:** Frances Yung, Jana Jungbluth, Vera Demberg

**Affiliations:** ^1^Department of Language Science and Technology, Saarland University, Saarbrücken, Germany; ^2^Department of Computer Science, Saarland University, Saarbrücken, Germany

**Keywords:** rational speech act model, discourse processing, discourse connectives, production, experimental pragmatics, crowdsourcing experiment, gamification

## Abstract

Rational accounts of language use such as the uniform information density hypothesis, which asserts that speakers distribute information uniformly across their utterances, and the rational speech act (RSA) model, which suggests that speakers optimize the formulation of their message by reasoning about what the comprehender would understand, have been hypothesized to account for a wide range of language use phenomena. We here specifically focus on the production of discourse connectives. While there is some prior work indicating that discourse connective production may be governed by RSA, that work uses a strongly gamified experimental setting. In this study, we aim to explore whether speakers reason about the interpretation of their conversational partner also in more realistic settings. We thereby systematically vary the task setup to tease apart effects of task instructions and effects of the speaker explicitly seeing the interpretation alternatives for the listener. Our results show that the RSA-predicted effect of connective choice based on reasoning about the listener is only found in the original setting where explicit interpretation alternatives of the listener are available for the speaker. The effect disappears when the speaker has to reason about listener interpretations. We furthermore find that rational effects are amplified by the gamified task setting, indicating that meta-reasoning about the specific task may play an important role and potentially limit the generalizability of the found effects to more naturalistic every-day language use.

## 1. Introduction

A speaker faces a number of choices when encoding a discourse relation: they can choose whether to leave it implicit, or mark the relation explicitly using a discourse connective. Discourse connectives (DC) are linguistic devices that signal coherence relations. Discourse theories such as the Rhetorical Structure Theory (RST; Mann and Thompson, 1988) distinguish between a large number of coherence relations and corresponding DCs; however, there is no one-to-one correspondence between them. One discourse relation can be signaled by multiple DCs, and one DC can signal a variety of different discourse relations. For example, a *causal* relation can be marked by *because* or *since*. In turn, *since* can signal a *causal* relation or a *temporal* relation about the starting point of an event.

The speaker thus often also needs to decide between several lexical alternatives for marking a specific discourse relation. The resulting variation in discourse connective choice is to date largely unexplained. We therefore here set out to test whether rational accounts of language processing, such as the uniform information density theory (Levy and Jaeger, 2007; Jaeger, 2010) or the rational speech act theory (Frank and Goodman, 2012) can account for this production choice.

These theories have been proposed to account for a wide range of phenomena in language production including speech articulation and the inclusion of optional syntactic markers (Jaeger and Buz, 2018), as well as referring expression production (Degen et al., 2013; Graf et al., 2016; Degen et al., 2020), omission of pronouns (Chen et al., 2018), ordering of adjectives (Hahn et al., 2018), and expression of exhaustivity (Wilcox and Spector, 2019). While the uniform information density hypothesis is most suitable for studying phenomena where the production variants are meaning-equivalent, the rational speech act theory also involves reasoning about alternative meanings of an utterance, and hence seems best suited for studying the production of discourse connectives. In fact, the RSA model has already been used to account for the distribution of explicit and implicit discourse connectives, and found it to be in line with the qualitative prediction of the RSA model (Yung et al., 2016, 2017). They found that, in the Penn Discourse Treebank (Rashmi et al., 2008), an explicit connective is more often omitted when the it is not informative enough to offset its production cost, or if there are enough other discourse signals in the arguments. The rational speech act theory (RSA) (Frank and Goodman, 2012; Goodman and Frank, 2016) is a formalization of Gricean pragmatics (Grice, 2000). It models and makes quantitative predictions on language production and comprehension in terms of a rational process by which speakers and listeners iteratively reason about each other. According to the RSA, a rational speaker aims at successful communication by calculating how the hearer would understand the speakers' utterance and choosing their utterance by trading off the likelihood that the utterance will successfully communicate the intended meaning against the speaker-effort of producing that utterance. Several variants of the RSA account have been proposed, including the *incremental* RSA (Cohn-Gordon et al., 2019), which allows the model to operate not only on the level of a sentence as a unit for defining successful communication, but holds that speakers may even aim to avoid temporary misunderstandings.

The current work thus seeks to find out whether the choice for a specific discourse connective is the result of a rational choice process in the speaker, who reasons about what discourse inferences the listener might make when hearing a specific discourse connective.

For example, a speaker might prefer the connective *whereas*, which signals *contrast*, over the connective *while*, which can signal both *contrast* and *temporal synchrony*, in a situation where the listener might be expecting a temporal relation, in order to direct listener expectations in the intended direction and avoid the risk of later misunderstandings. On the other hand, a speaker may well choose an ambiguous connective, if the intended coherence relation is easily predictable, and hence easy to disambiguate, by the listener.

Yung and Demberg (2018) set out to test whether connective choice in language production is a rational process as predicted by the RSA account, by setting up a language game experiment. In this experiment, the speaker is asked to express a target discourse relation to a listener by uttering a discourse connective, which either signals the relation unambiguously, or is ambiguous in that it can also signal other relations. The communicative utility of the connectives is determined by the set of possible interpretations that the listener might infer. These are shown explicitly to the speaker in the gamified setting used in Yung and Demberg (2018). In one case, both the ambiguous and unambiguous connective can safely be chosen to signal the relation, as no alternative interpretation that fits these connectives is part of the set of interpretations for the listener. In the other condition, the set of listener interpretations contains two relations that both fit the ambiguous connective. In this case, choosing the unambiguous connective is communicatively most useful, as it uniquely picks out the intended interpretation.

Yung and Demberg (2018) found that speakers in their experiment did choose the unambiguous DC option more often when the ambiguous option could fit with another given interpretation rather than the intended meaning, suggesting that people do reason about the comprehension of the listener. The results of that study were thus in line with the quantitative predictions of the RSA theory. However, there is an obvious gap between this gamified experimental design and naturalistic language use in communication: most importantly, the possible interpretations of a listener are normally not directly available to the speaker, but would have to be inferred. The prior study of Yung and Demberg (2018) thus only allows us to conclude that speakers CAN choose connectives rationally when they have the chance to reason about what the listener may understand, but does not show whether people actually DO make these rather complex inferences during normal language production. The question left unanswered is whether the explicit restriction on the valid interpretations, which only occurs in a gamified setup, is a critical factor that allows the speaker to reason about the listener's mind and make a rational choice, or whether the behavior found in Yung and Demberg (2018) also plays out in naturalistic language production. This is a concern that has been voiced also previously in the context of the RSA model: while the rational account allows to calculate what a perfectly rational speaker should do, there are concerns regarding the cognitive plausibility of the model (Borg, 2012; Carston, 2017; Borg, 2017): it is not always clear whether speakers actually make all those computations in real time every day language use. A specific contribution of this article from the point of view of rational models is that it does not approach this question by manipulating the necessary depth of reasoning or number of alternatives that need to be considered in reasoning, but investigates a case where reasoning needs to be done about an abstract object, namely coherence relations expected by the listener.

The current study aims to fill this gap by assessing the rational account of DC production under more realistic settings. In particular, we test people's discourse connective production choice in a setting where the possible interpretations of the listener are not limited to a specific set and are not explicitly available to the speaker. Instead, we manipulate discourse expectations of the listener, since people are sensitive to various signals in the context and build up expectation about the upcoming coherence relation (Lascarides et al., 1992; Kehler et al., 2008; Rohde et al., 2011; Rohde and Horton, 2014; Scholman et al., 2017, 2020; Schwab and Liu, 2020). In this more natural setting, showing that people prefer unambiguous DCs when there is a higher risk of misinterpretation by the listener (because the expectation is not in line with the actual ending) would substantially strengthen the empirical evidence for the rational account.

We here report on a series of experiments conducted via crowd-sourcing, where we manipulate what information regarding comprehender interpretation options is visible to the speaker. We replicate the effect found in Yung and Demberg (2018) when using the same game-like setup with explicitly given alternative continuations (section 2.4.3), but do not find any effect of discourse-related predictability on connective choice in our experimental settings where these continuations are not shown explicitly (section 3).

In section 4, we report on a follow-up experiment which tests whether the failure to find an effect of contextual constraint on connective choice is due to the lack of showing these alternatives, or whether it could be related to feedback during the experiment or other factors in the experimental setup which might encourage explicit reasoning about speaker interpretation.

Our results indicate that experimental design has a sizeable effect on connective choice—the game-like setting leads to more unambiguous connectives being chosen than more naturalistic designs. This brings up the question to what extent the results from gamified language tasks generalize to every-day language comprehension and production, or whether they are constrained to tasks that involve more explicit meta-reasoning.

This case study on DC production indicates that an easily calculable or explicitly available set of alternative interpretations is crucial for speakers to perform RSA-style reasoning. Overall, this study shows that accounts of rational language production might not be able to account for connective choice in everyday language communication.

### 1.1. Background

#### 1.1.1. The Rational Speech Act Theory

The rational speech act model (RSA) is a Bayesian computational framework based on Gricean pragmatic principles, which state that speakers try to be informative based on the knowledge shared with the listeners. Formally, given an intended meaning *m* to be conveyed, the pragmatic speaker in the RSA model chooses a particular utterance *u*′ from a number of alternative utterances that are compatible with meaning *m*. The probability that the speaker chooses *u*′ is proportional to the *utility* of *u*′ with respect to *m* and the shared background *C* (Equation 1). The *utility* of an utterance depends on the *cost* for the speaker to produce it and its *informativity*, which is quantified as the log probability of the listener inferring meaning *m* when they hear the utterance (see Equation 2). In the basic RSA model, the speaker reasons about a *literal* listener, who chooses an interpretation that is compatible with the utterance in context (Equation 3).

(1) $$\begin{array}{l} \begin{array}{ll} S_{prag}\left(u^{\prime }|m,C\right) & \propto e^{\alpha utility\left(u^{\prime };m,C\right)} \end{array} \end{array}$$

(2) $$\begin{array}{l} \begin{array}{l} utility\left(u;m,C\right)=\log L_{lit}\left(m|u,C\right)-cost\left(u\right) \end{array} \end{array}$$

(3) $$\begin{array}{l} \begin{array}{l} L_{lit}\left(m^{\prime }|u,C\right)\propto P\left(m^{\prime },C\right) \end{array} \end{array}$$

The *utility*, in the basic RSA model, is based on the comprehension of the listener after the complete utterance is processed.

The *incremental* RSA (Cohn-Gordon et al., 2019) further considers the informativeness of the incomplete utterance; it optimizes the utility of the next unit of production (e.g., word) where the context *C* is defined as the partial sentence uttered so far. Based on this modified version, speakers should choose their words such that temporary misunderstandings on the part of the listener are also avoided.

The RSA and other Bayesian rational accounts of language processing are supported by a set of experimental data on human communication, spanning a wide range of language phenomena (see Goodman and Frank, 2016 for an overview). A set of empirical study results can speak to the consideration of alternative interpretations and alternative utterances during language processing: it has been shown that the existence of alternative interpretations for an utterance affects the listener's processing of the actual utterance (Beun and Cremers, 1998; Bergen et al., 2012; Degen et al., 2013; Degen, 2013; Degen and Tanenhaus, 2015, 2016) and that speakers are sensitive to the informativity of referring expressions given the choices of objects in context (Olson, 1970; Brennan and Clark, 1996; Brown-Schmidt and Tanenhaus, 2006, 2008; Yoon and Brown-Schmidt, 2013). For example, while “*trousers"* is specific enough for the listener in a context containing a pair of jeans and a shirt, it would be ambiguous if there is also a pair of sweatpants. In turn, the speaker would avoid using the generic term “*trousers"* and prefer the more specific “*jeans"* to refer to the pair of jeans in the latter context. The availability of alternative utterances also matters. For example, Degen and Tanenhaus (2016) demonstrated that the processing of the scalar “*some"* is delayed in a context where the speaker is allowed to use exact numbers compared to when that option is not available.

The iterative reasoning between the speaker and listener proposed by RSA is in line with the literature on perspective-taking in the formulation and interpretation of utterances, which states that people generally take into account the knowledge and perspectives of their interlocutors (Stalnaker, 1978; Sperber and Wilson, 1986; Clark and Brennan, 1991; Clark, 1992, 1996; Pickering and Garrod, 2004; Barr and Keysar, 2005, 2006; Bard et al., 2000; Galati and Brennan, 2010; Pickering and Garrod, 2013; Ryskin et al., 2015).

For instance, a number of studies on referring expression production report that speakers generally adapt their production preferences to the knowledge of the listeners (Isaacs and Clark, 1987; Wilkes-Gibbs and Clark, 1992; Nadig and Sedivy, 2002; Yoon et al., 2012). Similar rational production behavior has also been found regarding the omission of pronouns when the referent is clearly understood (Chen et al., 2018) and the preference order of subjective adjectives (Hahn et al., 2018). One characteristic of the production scenarios that were examined is that the *intended meaning* is a concrete object with certain clearly distinguishable properties; the representation of its meaning does not require a high level of abstraction. It is as of yet unclear whether speakers can also reason about the informativity of an utterance when the meaning to convey is an abstract one, such as a coherence relation between segments of texts. We will discuss the evidence for comprehenders forming discourse expectations during comprehension below.

#### 1.1.2. Language Game Experiments

Many studies on RSA and perspective-taking make use of referential language games to test people's interpretation or production of referring expressions (e.g., Frank and Goodman, 2012; Degen et al., 2013; Vogel et al., 2014; Franke and Degen, 2016; Mozuraitis et al., 2018; Ryskin et al., 2015; Kreiss and Degen, 2020; Ryskin et al., 2020). Typically, in these game-styled experiments, a limited set of objects are presented to the participants, who are asked to interpret which object a particular referring expression refers to, or are asked to utter an expression to refer to a particular object. These studies typically show large RSA-consistent effects. This experimental paradigm defines toy worlds where the possible interpretations are limited to a controlled set of objects and allows the researchers to precisely manipulate the knowledge accessible to the speaker and the listener. For example, Ryskin et al. (2015) design privileged perspective where one interlocutor sees certain alternative objects in the context while the other does not. Despite these advantages related to experimental control, it has to be noted that these artificial settings are a simplification of the situation in the real world, where the possible interpretations are usually not limited, at least not as explicitly. In section 1.1.3, we will discuss the game-styled experiment presented in Yung and Demberg (2018), which captures the speaker's preference of DC choices when misinterpretation is (im)possible.

A more open-ended experimental environment is explored by a series of studies in Sulik and Lupyan (2016, 2018a,b). They use a signaling task where participants are asked to provide a single cue word to give a hint for the partner to guess a target word. In their setup, no alternative options of hints or target words are given, and the results show that the director uses salience information from their own perspective rather than that of the guesser. Participants' performance in choosing a cue word following the guesser's perspective can be improved with added contextual constraints and repeated interactions with feedback provided by the guesser, but further studies find that the improvement is based on other heuristics rather than better reasoning of their partner's perspective (Nedergaard and Smith, 2020). These findings suggest that people do not seem to reason about their listener's perspective in situations where the alternatives are completely unconstrained or unknown. However, the signaling game is a highly demanding task: it is not straightforward for the participant to come up with a cue from the guesser's perspective even if they actually try to do so.

These findings are consistent with studies in more complex perspective-taking settings, which suggest that there may be limitations to rational processing, showing that people do in fact often not behave optimally from the perspective of rational models. In tasks that require perspective-taking, i.e., when the speaker is aware that the information available to the listener is different from their own information, speakers tend to prioritize their own perspective when they are under time pressure (Horton and Keysar, 1996), or in situations where the information on their perspective is more salient (Lane and Ferreira, 2008). The recent study of Vogels et al. (2020) also found that speakers do not adapt their production to the cognitive load of the listeners on a fine-grained level, but rather adopt a very coarse strategy that they then follow: when the driver in a simulated driving task was under cognitive load, the speaker only made their utterances more redundant (easier to understand) if they had previously experienced the difficulty of the driver task themselves, and didn't adapt their strategy for trials where the cognitive load on the driver was lower.

In addition to the limitation on the alternative interpretations, the settings of game-styled experiments might entice people to engage in more extensive reasoning than usual, in order to guess the correct answer of the “riddle.” In particular, Sikos et al. (2019) found that, comparing with one-shot web-based experiments, increasing the participant's engagement in the task leads the participants to follow more closely to reasoning based on RSA, while the results of one-shot games are in some cases better fitted by simpler models based on literal interpretation (Qing and Franke, 2015; Frank et al., 2016; Sikos et al., 2019).

Taken together, results from referential language games show that people *can* reason about the reasoning of their interlocutors, but it is not clear if they would actually perform the same reasoning in everyday language use. Furthermore, prior findings indicate that speakers may not always have the capacity to behave optimally, even if they may strive to do so, and that they are happy to follow coarse heuristics for successful communication instead of reasoning on an utterance-by-utterance basis. A reason for this observation could be that maintaining a detailed mental model of the addressee's needs may be cognitively costly (Koolen et al., 2011; Horton and Keysar, 1996; Roßnagel, 2000).

#### 1.1.3. Language Game for DC Production (Yung and Demberg, 2018)

A gamified experimental design, similar to other RSA studies, is used in Yung and Demberg (2018) to compare the qualitative prediction of RSA against the choice of human subjects. The design adapts the language games of referring expression production for DC production. An example of the stimuli used is shown below.

**Example item from Yung and Demberg (****2018****)**:

That tennis player has been losing his matches…
  Options: *since / as / but*

**A. (Target production)… we know he is still recovering from the injury**.
B1. …the season started. /B2….he was close in every match.
C. …his coach believes that he still has chance.

In this experiment, the subjects act as speakers. They are given the first half of a sentence (*That tennis player has been losing his matches*) and a continuation which represents the speaker's communicative goal (continuation A: *.. we know he is still recovering from the injury*.) They are asked to choose one connective from one of the three given options (e.g., *since, as*, and *but*) to provide a “hint” to the other player regarding the intended target relation (*continuation A* as the target continuation out of three given continuations: A, B1 or B2, and C.

Furthermore, the speaker in the game can also see a set of alternative discourse continuations which the listener could possibly infer (continuation C and either continuation B1 or B2, depending on the condition). The subjects are told that the sets of connectives and continuations are also visible to the listener player, except that continuation A is the target.

The set of connectives and the set of discourse continuations are manipulated. Two of the connective options (*since* and *as*) can be used to mark the target relation (continuation A), but one of them is ambiguous (*since*, which fits both continuations A and B1) and the other is unambiguous (*because*, which fits only with continuation A).

The set of alternative continuations is set up such that it does or does not contain a continuation that is compatible with the other reading of the ambiguous connective (continuation B1, which matches the temporal reading of *since*). This manipulation of continuations thus creates a toy situation where mis-guessing is possible (including B1 in the alternative set), or not (including B2 instead of B1). Under this gamified setting, it was found that speakers do choose and unambiguous DC significantly more often in the former situation. In the current study, we are set to find out if the result still holds under a more naturalistic setting, where misinterpretation is manipulated by discourse expectation.

#### 1.1.4. Discourse Expectations

A variety of studies have shown that comprehenders use a range of cues to anticipate the continuation of the discourse (Sanders and Noordman, 2000; Rohde et al., 2011; Canestrelli et al., 2013; Köhne and Demberg, 2013; Rohde and Horton, 2014; Drenhaus et al., 2014; Xiang and Kuperberg, 2015; Scholman et al., 2017; Van Bergen and Bosker, 2018). Relevant cues include discourse connectives, as well as more subtle signals such as implicit causality verbs and negation. Köhne and Demberg (2013), for instance, found that people have different expectation about the upcoming discourse after reading a *causal* connective (e.g., *therefore*) vs. a*concession* connective (e.g., *however*). Similar results were also found in related studies such as Drenhaus et al. (2014), Xiang and Kuperberg (2015). People are also sensitive to more implicit signals apart from explicit connectives. For example, comprehenders anticipate a causal relation after encountering implicit causality verbs, such as *blame* (Rohde and Horton, 2014).

Apart from lexical signals, context is also considered to be important for the interpretation of discourse (Sanders et al., 1992; Lascarides et al., 1992; Cornish, 2009; Spooren and Degand, 2010; Song, 2010). Contextual signals that influence the expectation of a particular coherence relation are not limited to specific words that occur locally in the segments of texts joined by relation, but could locate in the more global context. For example, Scholman et al. (2020) showed that, in a story continuation task, people generate more *list* relations following a context where several similar events occurred, e.g., “*the woman experienced several unfortunate events last night. She got wine thrown at her by her dining companion…”*. However, the sensitivity to such contextual signal was shown to vary between different people: while some showed very high sensitivity, others seemed to ignore the signal, or not be able to take it into account Scholman et al. (2020). Furthermore, Schwab and Liu (2020) found that contrasting information in the context, e.g., “*he likes to run outdoors. He has a treadmill in the living room…”* facilitates the processing of a *concession* relation.

These works point to the fact that comprehenders generate expectation about the upcoming discourse continuation based on lexical and contextual cues in the preceding contexts. The current study aims at investigating the effect of contextual discourse expectation in combination with a rational account of connective production.

## 2. Material Construction and Methods

The objective of this study is to find out whether speakers choose discourse connectives rationally in a more naturalistic setting. Specifically, when the possible interpretations are not restricted and are not explicitly shown to the speaker, do speakers still reason about the listeners' difficulty in interpretation and prefer a disambiguating connective if the comprehender is likely to make the wrong inference?

In our experimental design, it is thus necessary to manipulate how easily the intended discourse relation can be inferred, without explicitly listing the possible interpretations. Our materials are constructed based on the strategy used in Yung and Demberg (2018). However, instead of limiting the possible interpretations allowed in the game, we propose to manipulate the interpretation difficulty by means of *contextual expectation*. We hypothesize that the target discourse relation is expected to be more difficult to infer in a context where a different coherence relation is expected, compared to a situation where the target discourse relation itself is highly expected. For instance, referring to the example presented in section 1.1.3, we create a contextual situation where the listener is expecting a reason (e.g., *The drop in performance of the tennis player was not coincidental. He has been losing his matches BECAUSE…*) or a specification of time (e.g., *Let me tell you how long that tennis player has been disappointing his fans. He has been losing his matches SINCE…*). The alternative interpretations, on the other hand, are not limited nor visible to the subjects. Following the prediction of the RSA model, speakers should use a more specific DC to express an unexpected discourse relation, while they may safely use an ambiguous connective if the target relation is already expected anyway. The construction of the stimuli will be explained in more details in the following subsection.

It is worth noting that the stimuli might not work properly if both meanings of the ambiguous connective are compatible with the target continuation. For example, in the sentence “*That tennis player has been losing his matches* ___________ *he changed his coach,”* the second clause can be read as a reason or a specification of time. The choice of *since* (ambiguous DC) instead of *ever since* (unambiguous DC for the temporal relation) may not be due to the adjustment in ambiguity level that we would like to test, but rather because the reason reading is preferred by the subject. In other words, the alternative relation senses of the ambiguous DCs elicited by the stimuli have to be distinctive enough. To verify this, we conducted a pretest on the stimuli on another group of subjects. The details will be explained in section 2.4.1.

Another necessary verification of the experimental materials is to test whether the situational contexts of the items do increase the expectation of a particular discourse relation as we expect in the design. Section 2.4.2 describes the pretest we carried out to verify this. Finally, the newly constructed stimuli should also work in the gamified setting. We thus try to replicate the results of Yung and Demberg (2018) with the new set of stimuli in our third pretest (section 2.4.3).

### 2.1. Stimuli Construction

The pattern of our experimental stimuli is as follows: We determine two alternative discourse relations, the target relation (TR) and the competitor relation (CR). Next, we select a pair of connectives such that one of the connectives is ambiguous and can signal both TR and CR, while the other connective is unambiguous and can only signal TR. We then need to design a discourse relational argument Arg1 which is compatible with either relation, and two continuations, one conveying the target relation, and the other conveying the competitor relation. We denote these relational arguments as TA and CA respectively. Finally, in order to manipulate which of the coherence relations is expected, we construct two different contexts that raise discourse expectations for each of these relations. As a baseline, we also add a neutral context, and a third unrelated connective which marks neither TR nor CR. An example of an item is given in Table 1.

**Table 1:** Material construction pattern illustrated with a concrete example.

1a	Context for TA	*Chris is a professional artist and so is his wife. However, his talent is very different from hers:*
1b	Context for CA	*I am going to the music festival with my friends next week. I look forward to a particular performance by a musician who can play two instruments at the same time:*
1c	Neutral context	*I had a very nice lunch with my old friend Chris today. I haven't seen him in a long time. Chris loves music:*
2	Arg1	*he plays the saxophone*
3	Connective choice	*while* (TA / CA), *whereas* (TA), *specifically* (other)
4a	TA (target Arg2)	*his wife is a ballet dancer*
4b	CA (competitor Arg2)	*he accompanies himself on the drums*.

*TA stands in a contrastive relation to Arg1 in this example, while CA stands in a temporal-synchronous relationship with Arg1*.

In this example, the target discourse relation to be produced is a contrast relation, between “*he plays the saxophone”* and “*his wife is a ballet dancer.”* We call “*he plays the saxophone”'* the *first argument*, abbreviated Arg1 of the discourse relation, and “*his wife is a ballet dancer”* the *target second argument* (TA). The TA is a specific instantiation of the abstract relation type to be produced by the speaker, and connectives that mark the relation type are provided as options for the speaker to choose from. Among the provided options, both *while* and *whereas* mark a contrast relation, but *while* is more ambiguous because it can also mark the temporal relation between two events happening at the same time. On the other hand, *and specifically* does not fit the *target continuation*. We call *while, whereas* and *and specifically* the *ambiguous, unambiguous* and *incompatible* DC respectively. The *incompatible* DC is chosen such that the relation it signals is considerably different from any of the relations signaled by the *ambiguous* and *unambiguous* DCs.

In our experiment, the speaker will see one of the contexts (1a, 1b, or 1c) and the first argument (2), and will be asked to choose among the three connectives (3). The speaker will also see the intended second argument (4a). The competitor second argument (4b) will never be shown, it thus remains implicit. We constructed a total of 62 items following this pattern.

According to the RSA, it is rational to prefer the unambiguous connective *whereas* over the ambiguous connective *while*, especially in a context where the competitor argument (CA) is contextually expected: selecting the ambiguous connective which is compatible with CA would leave the comprehender on the wrong track and lead to difficulty in inferring the correct continuation TA.

In order for the stimuli to work in the intended way, it is important for the two coherence relations that are marked by the ambiguous connective to be distinct from one another, such that the unambiguous connective intended to mark only the target relation TR is not compatible with the competitor relation CR. We therefore selected three connectives, *since, as*, and *while* as the ambiguous connectives in our experiment, as they each signal two relations that are distinct from one another. Table 2 summarizes the target discourse relations and the DC options covered by the stimuli. The intended mismatch between the unambiguous connective and the competitor second argument is tested in our first pretest, see section 2.4.1.

**Table 2:** Summary of the stimuli.

**Ambiguous DC**	**Target discourse**	**Unambiguous DC options**	**Stimulus count**
	**relation**		
*since*	causal	*because*	10
*since*	precedence	*ever since*	10
*as*	causal	*because*	10
*as*	synchronous	*when, while, at the same time as, etc*.	10
*while*	contrast	*whereas, but*	11
*while*	synchronous	*when, as, during the time when, etc*.	11

*The incompatible DCs used in the stimuli include if, unless, in other words, for example, so that etc*.

### 2.2. Participants

All pretests and experiments reported in this article were conducted online via the crowd-sourcing platform Prolific. Participants were restricted to English native speakers currently residing in English-speaking countries. Also, only participants with past approval rates of 99% or more were selected. Details on the participants will be reported in each specific experiment.

### 2.3. Procedure

In the beginning of the experiment, the participants were informed that collected data will be used for research purposes and that all data will be anonymized prior to analysis. They were also informed that there are no risks or benefits to participating in the study and their contribution is voluntary, and thus they might decline further participation, at any time, without adverse consequences. The participants' consent and confirmation of being at least 18 years old were obtained before the start of the experiment.

### 2.4. Norming and Pretests

We conducted three pretests to make sure that our stimuli work as intended. The first pretest was run to validate whether the unambiguous connective is indeed incompatible with the competitor relation. This pretest is reported in section 2.4.1 below. The second pretest aims at testing whether the biasing contexts 1a vs. 1b indeed raise different discourse expectations (4a vs. 4b), and is reported in section 2.4.2. Finally, we repeated the experimental setup described in Yung and Demberg (2018) with our new materials, in order to check whether we can replicate their results (section 2.4.3).

#### 2.4.1. Pretest 1: Validation Relation Interpretations

One difficulty in stimulus design is that the relations themselves can sometimes be ambiguous. In those cases, a participant might infer both readings, or the participant may only infer one reading, but we don't know ahead of time which one. Both of these cases are problematic.

For an example of a case where the participant may infer both readings, consider the sentence *John started to clean his flat regularly* ***since****his girlfriend moved in*. In this example, *his girlfriend moved in* could be the reason, or just the marker of the specific time. Both of the unambiguous markers (*because* for the causal reading and *ever since* for the temporal reading) would in that case be compatible with the continuation, and hence there would be no rational advantage to choosing the unambiguous connective over the ambiguous one.

If, on the other hand, a participant only infers one of these relations, we also have a problem because we don't know ahead of time which one it will be and what connective we should hence provide as the unambiguous alternative. For instance, if the participant interprets *his girlfriend moved in* as the continuation of a temporal relation (the CR), then *because* is no longer a valid marker for the TR in the stimulus. Hence, we do not want to include sentences where both the TR and CR are possible.

The objective of this pretest is thus to confirm that the target continuation of each stimulus represents a discourse relation that is highly distinguishable from the competitor discourse relation. Accordingly, the acceptability of each connective option is tested with respect to the intended discourse relation.

##### 2.4.1.1. Materials and Procedure

The pretest was carried out in the form of a coherence rating task. We created two sentences for each experimental item by inserting the unambiguous connective and an unambiguous connective expressing only the competitor relation between the first argument and target second argument as shown in the following example.

**Stimulus item:**

James has been studying very hard ___________ he entered secondary school 2 years ago.
  (Options: *since, ever since, instead*)

**Pretest items:**

1. connective compatible only with TA:
  James has been studying very hard **ever since** he entered secondary school 2 years ago.
2. connective compatible only with CA:
  James has been studying very hard ^*^because he entered secondary school 2 years ago.
3. ambiguous connective:
  James has been studying very hard **since** he entered secondary school 2 years ago.
4. incompatible connective:
  James has been studying very hard ^*^instead he entered secondary school 2 years ago.

Participants were asked to rate the coherence of each pretest item on a scale of 1 (least acceptable) to 4 (most acceptable). They could also optionally suggest a word or phrase to replace the bold DC to improve the acceptability of the sentence. This additional feedback provided suggestions for the improvement of the stimuli. Since the focus of this pretest is the discourse relation between the first argument and the target continuation, preceding contexts are not included in the pretest items.

For items that work as intended, variant (1) with the unambiguous connective from the original item should be judged to be substantially better than variant (2). Furthermore, variant (3) verifies if the ambiguous connective fits the original item and variant (4) confirms the incompatible connective is not acceptable. Hence, variant (3) should be judged with high ratings while variant (4) should be rated worse.

##### 2.4.1.2. Participants

The items were distributed evenly across 16 lists, and each list was completed by 15 participants. Each participant only saw one version of an item. They also did not see items sharing the same first arguments. A total of 411 participants (age range: 20–75, mean age: 36, 257 females) took part in several rounds of the pretest. They were recruited via the crowdsourcing platform Prolific according to the criteria described in section 2.2.

##### 2.4.1.3. Analysis

We define the *semantic gap between alternative discourse relations* of a stimulus based on the difference in the average rating of the intended and unintended version of the pretest item. For example, the average ratings of pretest items 1 and 2 shown above were 3.87 and 2.27, respectively. The *semantic gap* is thus 3.87 − 2.27 = 1.60, which can be normalized to 53% based on the maximally possible difference of 3. Stimuli with semantic gap below 5% were replaced or revised. The revised stimuli underwent another round of pretest. Several rounds of pretests were conducted on several subsets of the items until the semantic gaps of all items were above 5%. The results of the final version of the items were collected from a total of 360 participants.

##### 2.4.1.4. Results

The average coherence rating for the final items was 3.47 for the variant with the unambiguous connective fitting with target second argument TA, and 1.59 for the unambiguous connective that fits the competitor second argument CA. Ambiguous DCs and incompatible DCs received average ratings of 3.15 and 1.25, respectively. The semantic gap between final versions of the stimuli ranged between 5 and 96%, with an average of 62%.

#### 2.4.2. Pretest 2: Validation of Target- and Competitor-Predicting Contexts

The second pretest is performed to confirm the contextual conditions of the stimuli. Referring to the example shown in Table 1, we want to make sure that the target-predicting contextual condition (1a) raises the prediction for a contrastive relation and fits together with the target relational argument (4a). On the other hand, the competitor-predictive context (1b) should be predictive of a temporal synchronous relation and should fit with competitor continuation (4b), but not vice versa.

##### 2.4.2.1. Materials and Procedure

The pretest was formulated as a forced choice task in which participants were asked to select the discourse continuation that best fit the context, see the following example:

**Pretest items**:

(1a) Context A, here contrast-predicting context:
  Chris is a professional artist and so is his wife. However, his talent is very different from hers: he plays the saxophone
(1b) Context B, here synchronous-predicting context:
  I am going to the music festival with my friends next week. I look forward to the particular performance by a musician who can play two instruments at the same time: he plays the saxophone
(2a) Continuation fitting Context A, here contrast:
  …whereas his wife is a ballet dancer.
(2b) Continuation fitting Context B, here synchronous:
  …at the same time as he accompanies himself on the drums.

The order of the two options was randomized in the study.

##### 2.4.2.2. Participants

The items were distributed evenly among 9 lists, such that each item was responded to by 15 participants. Like in the previous pretest, each participant only saw one condition of each item. Across several rounds of pretests, we recruited a total of 263 participants (age range: 22–74, mean age: 36, 188 females) via Prolific, based on the same criteria as mentioned above, and excluding participants who had taken part in the previous pretest.

##### 2.4.2.3. Analysis

We define the *contextual gap* between target- and competitor-predicting contexts based on the difference in the number of participants choosing the matching vs. non-matching continuations. For example, 14 participants chose continuation (2a) when given context (1a), and 0 participants chose continuation (2a) when given context (1b). The score of *contextual gap* of this stimulus pair is thus 14 − 0 = 14, which can be normalized to 93% based on the possible range of 0 − 15. Stimulus pairs with a contextual gap below 25% were replaced or revised.

Several rounds of pretests were conducted such that the final version of the items all have a contextual gap larger than 25%. The results of the final version were collected from 135 participants.

##### 2.4.2.4. Results

The mean number of votes of the expected and unexpected relations are 12.48 (SD=2.51) and 2.52 (SD=2.51) respectively, showing that the situational contexts used in the stimuli do trigger the expectation of one discourse relation in comparison to the alternative relation signaled by the ambiguous DC. The average contextual gap for the final stimuli was 68%, ranging from 27 to 93%.

#### 2.4.3. Pretest 3: Replication of Yung and Demberg (2018)

The final pretest aims at verifying whether the created stimuli can elicit pragmatic inference under setting used in Yung and Demberg (2018), where the alternative Arg2 continuations are shown to the speaker explicitly.

##### 2.4.3.1. Materials and Procedure

As contextual prediction is less relevant when the alternative continuations are presented explicitly, we performed this pretest using the *neutral* contexts. The speaker is shown the context, a choice of three connectives, and a set of three alternative second arguments. The speaker is told that the listener will have to guess which argument is the correct continuation, based on the connective that the speaker provides as a cue.

The three alternative second arguments consist of the target argument TA (continuation A in the below example), the competitor argument CA (continuation B1 below) and an unrelated completion C which is linked to the first argument via a different coherence relation. The target argument TA is indicated to the speaker by bold font. The experimental condition displaying options A, B1, and C corresponds to the CA-predictive context, for which a rational speaker should prefer the unambiguous marker *whereas* to mark relation A. A second condition in this experiment consists of continuations A, B2, and C. This condition corresponds to the TA-predictive context; here, both *while* and *whereas* signal relation TA unambiguously, therefore, the choice between them doesn't matter in this condition. Here is an example of the items used in the pretest.

**Pretest 3 item**:

I had a very nice lunch with my old friend Chris today. I haven't seen him in a long time. Chris loves music: he plays the saxophone…

Options: *while / whereas / specifically*

**A. … his wife is a ballet dancer**.
B1. (with competitor) … he accompanies himself on the drums.
B2. (no competitor) … he plays it every evening after dinner.
C. … he is good at playing jazz.

We also constructed filler items, which had the same format as the test items, except that the target was continuation B or C. In the fillers, only one of the provided DCs thus fit the target continuation. In the example, only *while* fits continuation B1 and only *specifically* fits continuations B2 and C.

The second player was programmed to be a rational listener, i.e., the simulated hearer would choose continuation A if the speaker selected the unambiguous connective, and continuation B if the speaker selected the ambiguous connective. In the unambiguous condition, the simulated player was programmed to choose option A for both connectives. The participant received one point if the guess of the hearer was correct. At the end of the experiment, bonuses were issued based on the total points. The bonus system encourages participants to engage more in the communicative task.

The items were evenly distributed into 12 lists. Each list contained 10-11 items and fillers. The conditions, discourse connectives and relation types were fully counterbalanced. The target continuation was always presented to the speaker as continuation A, while the other two alternative continuation were randomly assigned to B and C. The order of the three discourse connectives was also randomized per participant.

##### 2.4.3.2. Participants

We recruited 180 participants (age range: 19–71; mean age: 34; 99 females) via Prolific under the same criteria as the other studies, excluding participants who had taken part in the previous pretest. Participants who chose 4 or more non-matching connectives were replaced.

The participants were assigned evenly to the 12 lists; each participant saw 10-11 experimental items and 10-11 fillers.

##### 2.4.3.3. Analysis

We analyzed the data using a Binomial Liner Mixed-Effects Regression Model (*lme4* implementation in R, Bates et al., 2015), with connective choice as a response variable and continuation set as a predictor. The unambiguous DC was coded as 1, and the ambiguous connective as coded as 0. The models reported below include random intercepts by participant, as well as random intercepts and slopes for continuation set by item. Random slopes by participant had to be removed since they couldn't be effectively estimated by the model (their random effects correlation was 1.0).

##### 2.4.3.4. Results

The linear mixed effects analysis reveals a significant effect of condition (what options are shown as possible continuations) on connective choice β = 0.560; *SE* = 0.138 *z* = 4.049, *p* < 0.001. This finding is consistent with the results by Yung and Demberg (2018) and indicates that the presence of a competitor relation in the alternative options increases the preference for the unambiguous connective.

**Figure 1:**
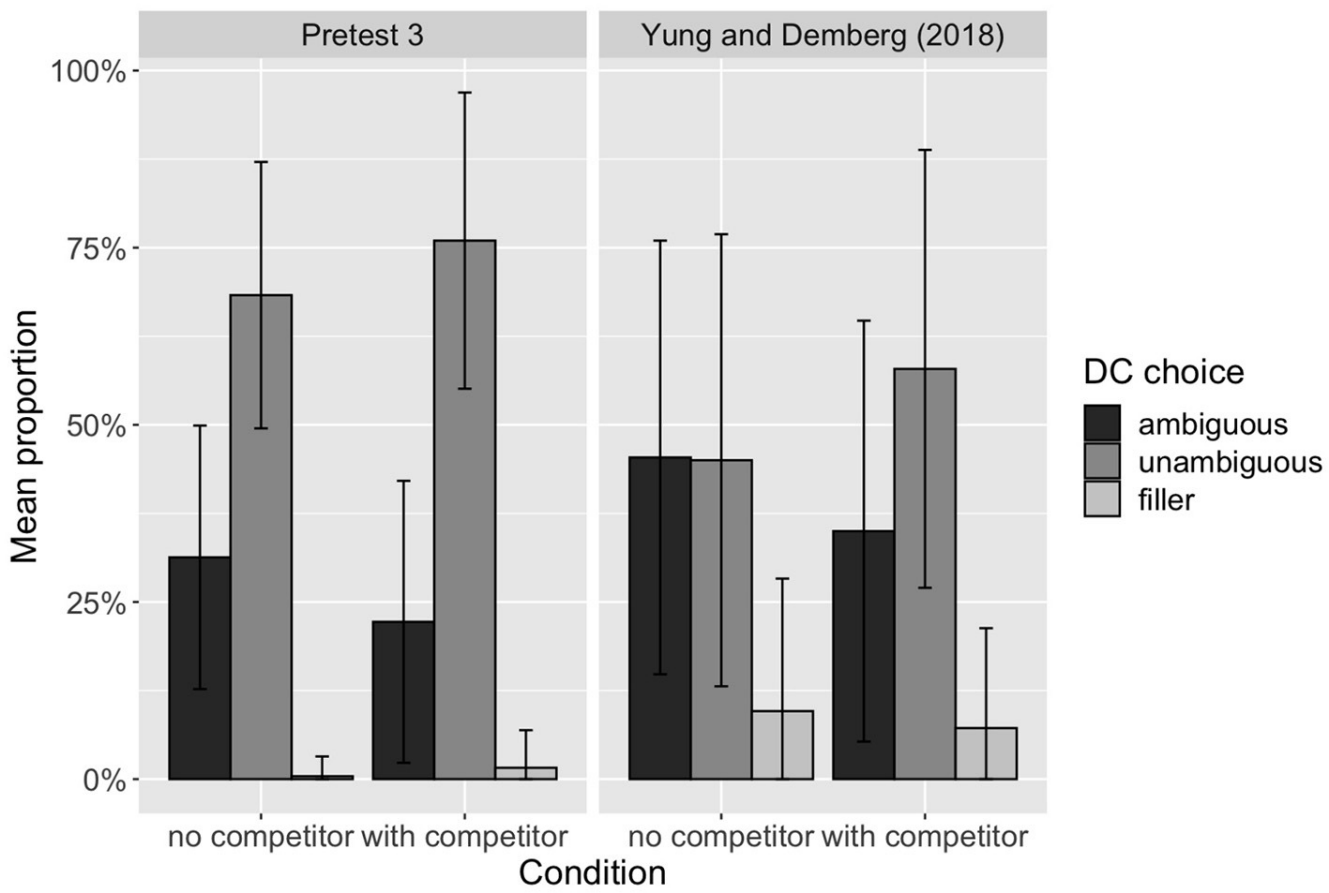
Distribution of the proportion of DC choices made by each participant in the language game experiment of Pretest 3 and Yung and Demberg (2018).

Figure 1 compares the results of this pretest with those from Yung and Demberg (2018). While Yung and Demberg (2018) found that speakers did not have a preference between the ambiguous and unambiguous DCs in the *no competitor* condition, the results for our new items show a general preference for the unambiguous DC, even when there is no ambiguity.

##### 2.4.3.5. Discussion

We believe that this discrepancy in results can be attributed to the differences in the stimuli we use: our stimuli include a different distribution of ambiguous DCs and their unambiguous alternatives compared to Yung and Demberg (2018). For example, the unambiguous DC *because*, which is a very frequent marker, is used in our stimuli as the unambiguous option for a *causal* relation, but it is not included as an option in Yung and Demberg (2018). On the other hand, the ambiguous DC *while* is frequently used to mark the synchronicity of two continuous events, while the unambiguous version *at the same time as* is much rarer. This highlights the importance of experimental control over other factors of DC production, such as frequency.

We therefore also included connective identity as a predictor in the model, and found significant differences between the connective pairs with respect to how likely the unambiguous connective was to be chosen by the participants (*since*: β = 0.905; *SE* = 0.347 *z* = 2.609, *p* < 0.01; *while*: β = −0.949; *SE* = 0.320 *z* = −2.963, *p* < 0.01). These differences did however not change the overall effect of the presence of a competitor second argument on connective choice.

Overall, the pretest results confirm that this set of stimuli can elicit RSA-like rational DC production, in a language game setup where the alternative interpretations are restricted. We next proceed to examine whether similar results can be produced in a more natural setup, i.e., when the possible interpretations are not restricted.

## 3. Experiment 1: Speaker's Choice of DCs When the Interpretations Are Unrestricted

The objective of this work is to examine whether the explicit availability of the comprehensible discourse relations—an artificial situation presented in a language game experiment—is a crucial factor for speakers to rationally choose a connective. To this end, in our first experiment, we replace this experimental design choice by creating an “invisible” set of alternatives based on the contextual predictions which should lead the comprehenders to expect a specific discourse relation, even if it is not explicitly shown. Our goal is to test whether the speaker's preference between an ambiguous and unambiguous DC shows the same tendency as in the language game experiment, is observed.

In this experiment, the possible continuations of the discourse are neither restricted nor explicitly defined—a situation that resembles natural communication more closely. To assess the rational account of connective production, it is however necessary to create a condition where mis-interpretation is predicted, or not, by the speaker. Such manipulation is achieved in the language game design by including a competitor or not in the available interpretations.

Here, we create two contrasting conditions that correspond to the *with* and *without competitor conditions* by manipulating the preceding contexts without restricting the interpretations, as described in section 2.1. A context where the *target discourse relation* is expected corresponds to the *without competitor condition*, as mis-comprehension is less likely. In contrast, the listener may fail to interpret the target relation when *the competitor relation* is contextually expected, and this condition corresponds to the *with competitor condition*. Following the qualitative prediction of RSA, we expect that speakers will choose the unambiguous connective more often when the preceding context elicit the expectation of the competitor relation.

### 3.1. Procedure and Materials

The 62 stimuli described in section 2.1 were split into 15 lists, each containing 12 stimuli, such that stimuli sharing the same first argument were never included in the same list. The types of ambiguous DCs, target discourse relations and experimental conditions were counterbalanced. Each list also contained 12 filler items which were taken from a total pool of 18 unique fillers. The items and options were presented to each participant in random order. The fillers have the same structure as the actual stimuli, but are always unambiguous. The purpose of the fillers is to avoid expectation from the participants that there are always two correct options per question. The fillers also help us in screening spammers who answer randomly.

The participants were instructed to imagine that they were reading the sentences to a friend over the phone, but one of the words was blurred and illegible, and they should choose a word from the options to replace it.

### 3.2. Participants

Two hundred and twenty-five native English speakers (age range: 19–70, mean age: 38, 125 females) were recruited via Prolific.ac. 144 of them reside in the U.K, 54 in the U.S. and the rest in Australia or Canada. They did not take part in any of the pretests. They took an average of 10 min to finish the task and were awarded 1.34 GBP for their contribution. 16 workers who had more than 10% wrong answers (choosing a DC that does not match the target continuation) were removed and replaced.

### 3.3. Analysis

We used a binomial linear mixed effects regression model to analyze the effect of the three contextual conditions on DC choice. Again, the unambiguous connective was coded as 1 and the ambiguous connective as 0. Context type was dummy coded, with the competitor predicting context as the base level. Random by-participant and by-item intercepts as well as by-item slopes for the contextual condition were included. We furthermore included semantic gap and contextual gap, which were estimated as part of pretests 1 and 2, as covariates in the model, to account for differences between the items. Responses choosing the *incompatible* DCs were not taken into account. Additionally, we also performed a Bayes Factor analysis using full Bayesian multilevel models. The Bayesian inferences were done using Markov Chain Monte Carlo (MCMC) sampling with 4 chains, each with iter = 6,000; warmup = 1,000; thin = 1; post-warmup = 20,000. The models were implemented using the BRMS package in R (Bürkner, 2017). We here report results for the default prior, which is an improper flat prior over the reals. For the effects that were not found significant in the linear mixed effect model, we report the Bayes factor expressed as BF_01_, indicating the odds for the null hypothesis H0 compared to the H1 based on the data.

### 3.4. Results

The binomial linear mixed effects regression model showed no difference between the competitor-biasing context condition and the target-biasing context condition (β = −0.022; *z* = −0.184, *p* > 0.05), and also no significant difference between the competitor-biasing context and the neutral context (β = −0.123, *z* = − 0.958, *p* > 0.05), see also Table 3.

**Table 3:** Regression coefficients of the binomial linear mixed effects model for Experiment 1.

**Variable**	**β**	**SE**	**z**	**p**
Intercept	−0.006	0.765	−0.008	0.994
Target-predicting context	−0.022	0.118	−0.184	0.854
Neutral context	−0.123	0.128	−0.958	0.338
Semantic gap	1.755	0.694	2.529	0.011^*^
Contextual gap	−0.395	0.786	−0.502	0.616

* *p < 0.05*.

We therefore also ran Bayesian multilevel models. Their results were consistent with the results of the linear mixed effects models, and showed no effect of context (target-biasing context: *t* = −0.03; 95% *CI* [−0.26, 0.21], neutral context: *t* = −0.13; 95% *CI* [−0.39, 0.13]). The Bayes Factor (*BF*_01_) comparing the reduced model without context as predictor (H0) to the model including context as predictor (H1) is 32.88, indicating very strong evidence in support of H0.

**Figure 2:**
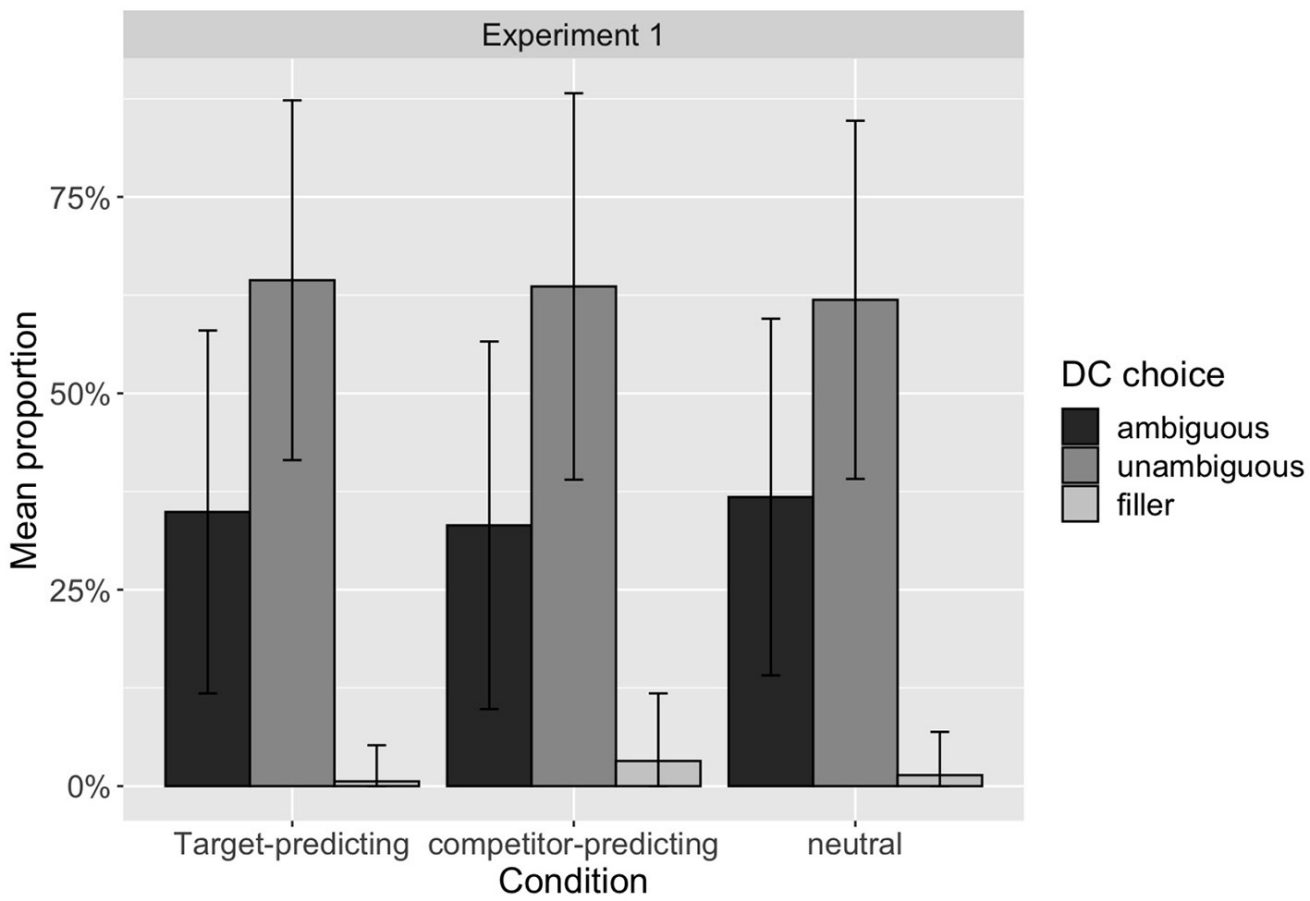
Distribution of proportion of the DC choices made each participant in Experiment 1.

The lack of effect is also visualized in Figure 2, which displays the proportion of connective choices in the three conditions. Excluding the small number of choices of the incompatible DCs, which can be interpreted as the cases where the participants were not producing the intended target discourse relation, the proportion of unambiguous DC choices are similar, namely 65, 66, and 65% under the *target-predicting, competitor-predicting*, and *neutral* conditions, respectively. In contrast to our hypothesis, the *competitor-predicting* condition does not increase the speaker's preference to use an unambiguous DC.

We furthermore find a statistically significant effect of semantic gap on connective choice, see Table 3. Items with a large semantic gap between the alternative discourse relations result in a larger proportion of unambiguous DC production compared to items with a smaller semantic gap.

This effect indicates that the unambiguous connective was preferred when the unambiguous connective could clearly not mark the competitor continuation. There was no effect of contextual gap (this is an expected outcome given that the contextual conditions do not affect the DC choice). The interactions between contextual gap and context type [χ^2^(2) = 2,105, *p* > 0.05], or between semantic gap and context type [χ^2^(2) = 2.194, *p* > 0.05] did not improve model fit.

### 3.5. Discussion

The experiment results suggest that the expectation of the forthcoming discourse relation to be produced does not affect the speaker's choice of discourse connective. This means that contextual expectation of the competitor discourse relation does not specifically trigger speakers to use an unambiguous DC to encode the target relation, while explicitly displaying the competitor continuation does, as shown in Pretest 3. A possible explanation would be that people perform RSA-style reasoning only in a game setting, where (i) meta-reasoning about what the listener will choose as a coherence relation is encouraged, and where (ii) reasoning about listener interpretation is facilitated by explicitly showing the alternative interpretations, i.e., this inference does not have to be performed by the speaker, and by rewarding the speaker if the listener would guess correctly. It is thus possible that this setup encouraged deeper reasoning about the task, or facilitated learning: when the rational listener gave a non-target response, the speaker may have used this feedback to adapt their strategy and subsequently avoid ambiguous connectives.

We therefore conducted a follow-up experiment in which we still do not show the alternative possible interpretations by the listener, but try to encourage meta-reasoning to a similar extent as in pretest 3.

## 4. Experiment 2: Investigating the Effect of Experimental Design

Experiment 1 and pretest 3 yielded different results (pretest 3 was consistent with the RSA hypothesis, while experiment 1 was not). These experiments however differ in two ways: Firstly, pretest 3 explicitly lists the different listener interpretations, while experiment 1 manipulates discourse expectations, without explicitly showing what the discourse expectations are; secondly, the experiments also differ in terms of setup and instructions, specifically, the instructions of pretest 3, which ask the participant to provide the connective cue in order for the listener to guess the correct second argument, might entice participants more strongly to perform meta-level reasoning to gain points in the game, while experiment 1 uses a more naturalistic situational setting.

Experiment 2 thus aims at teasing apart these two factors. We do this by designing the instructions to match the instructions of pretest 3, while still not showing the alternative listener interpretations to the speaker. A comparison between experiments 1 and 2 will then allow us to investigate whether the lack of effect in experiment 1 can be attributed to the difference in study instructions. To this end, we run the first half of the experiment just like pretest 3, thus providing the participants with training and the mindset of pretest 3. We then add a novel condition in the second half of the experiment. In this novel condition, the speaker sees three alternative listener interpretations, but only one of them (the target) is readable, while the other two are blinded, see bottom right cell in Table 4. In experiment 2, we again use the three contexts (target-predicting, competitor-predicting and neutral), and balance them across all conditions. Note though that we do not expect an effect of context in the first half of the experiment—here the listener interpretations are shown explicitly and hence overrule any expectations about listener inferences. We do however expect an effect of context in the second half of the experiment, where the alternative continuations are not readable and hence need to be “instantiated" by the speaker based on the predictions derived from the context.

**Table 4:** An example of a stimulus in various conditions.

**1. Preceding context:**		
**Target-predicting condition^1,2n,2w^**	**Competitor-predicting condition^1,2n,2w^**	**Neutral condition^p3,1,2n,2w^**
Chris is a professional artist	I am going to the music festival with	I had a very nice lunch with
and so is his wife. However, his	my friends next week. I look forward	my old friend Chris today.
talent is very different from hers:	to the particular performance by a	I haven't seen him in a long
	musician who can play two instruments	time. Chris loves music:
	at the same time:	
**2. Core stimulus: first argument and connective choices^p3,1,2n,2w^:**		
he plays the saxophone ___________ (while / whereas / and specifically,) …		
(*while*=ambiguous DC, *whereas*=unambiguous DC, *and specifically*=incompatible DC)		
**3. Target and alternative continuations**:		
**No competitor condition^p3,2n,2w^**	**With competitor condition^p3,2w^**	**Blinded condition^1,2n,2w^**
between semantic A. his wife is a ballet dancer…	A. his wife is a ballet dancer…	A. his wife is a ballet dancer…
B2. he plays it every evening	B1. he accompanies himself on	B. ■■■■■■■■■■■■
after dinner…	the drums…	
C. he is good at playing jazz…	C. he is good at playing jazz …	C. ■■■■■■■■■■■■
		

*For comparison, experiments including the corresponding conditions are indicated: p3, Pretest 3; 1, Experiment 1; 2n, Experiment 2 (no pragmatic exposure); 2w, Experiment 2 (with pragmatic exposure)*.

In summary, the most interesting part of the second experiment is its second half: here, the participants have all the instructions and experience just like in pretest 3, but cannot see the alternative continuations, like in experiment 1.

In addition, we want to evaluate how the language game experience in the first half would affect people's performance under the blinded-continuations-condition. Specifically, do people consider the potential risk of ambiguity in the connective, if they haven't seen any effects of ambiguity earlier? And do people adapt their choice based on feedback during the first half of the experiment, such that an incorrect guess by our rational listener may entice the speaker to subsequently prefer the unambiguous connective. To test whether there is such an effect of language game experience, we therefore introduce two training conditions in the first half of the experiment: in the one condition, the alternative continuations explicitly shown to the participants include competitor continuations, and the feedback is from the rational listener, just like in pretest 3, while in the other condition, the participants never see any competitor continuations in the first half, and feedback comes from a literal listener, thus avoiding to give feedback that may specifically encourage rational behavior.

This study will help to shed light on the effect of task formulation on rational reasoning effects in experimental studies.

### 4.1. Materials

We again use the materials as described in section 2, but add a *blinded* condition. The blinded condition is designed to resemble the situation where the possible discourse continuations are unlimited, because it does not provide any information of the alternative continuations. Table 4 provides an overview of the conditions of all experiments.

### 4.2. Procedure

The experiment is based on the language game design used in Pretest 3 (section 2.4.3) that manipulates the alternative continuations, except for the following modifications:

1. Instead of using the *neutral* context in all items, *target-predicting* and *competitor-predicting* contexts are also included as experiment conditions, and are counterbalanced with the *no* and *with competitor* conditions.
2. Each task to be finished by one participant is divided into two halves. In the first half of the task, the alternative continuations are always shown to the participants. For half of the participants, the setup is the same as in pretest 3, for the other half, only the unambiguous condition is included, in which there is never a competitor second argument. In the second half of the task, however, only the target continuation is shown, and the alternative continuations are *blinded*, i.e., NOT readable to the participants. An example is shown in the bottom right corner of Table 4.
3. Each task is implemented in two different versions, which we call the *with* and *without pragmatic exposure* versions respectively. The two versions differ in whether the *with competitor* condition is included or not. In the first half of the *with pragmatic exposure* version, half of the stimulus items have a competitor in the given alternatives, just as in Pretest 3, while in the *without pragmatic exposure* version, there are never competitors in the alternative continuations.

To summarize, the first half of the *with pragmatic exposure* version is a 3 × 2 design (*target-predicting/competitor-predicting/neutral* by *with competitor/no competitor*), while the first half of the *without pragmatic exposure* version is a 3 × 1 design (3 contextual conditions by *no competitor*). The second halves of both versions also have 3 conditions (3 contextual conditions, with *blinded* continuations).

Note that the feedback provided by “Player 2” (the listener) is also programmed differently in the two versions. “Player 2” of the *with pragmatic exposure* version reasons about Player 1's choices and answers rationally, while “Player 2” of the *without pragmatic exposure* version will correctly guess the target as long as it's compatible with the chosen connective. Although the alternative continuations are blinded, the guesses made by “Player 2” are shown to the participants as feedback. In the *without pragmatic exposure* version, “Player 2” never guesses a competitor while in the *with pragmatic exposure* version, a competitor is always returned as a feedback whenever an *ambiguous* DC is chosen. An overview of the experimental design is provided in Table 5.

**Table 5:** Task structure of Experiment 2.

	***With pragmatic exposure* version**	***Without pragmatic exposure* version**
1st	**12-13 stimuli**: the target continuation matches **both the ambiguous and unambiguous DCs**	
half	Contextual condition: target-predicting / competitor-predicting / neutral (counterbalanced)	
	Alternatives: no / with competitor (counterbalanced)	Alternatives: **no competitor only**
	**Rational feedback**: “Player 2” guesses the competitor	**Literal feedback**: “Player 2” guesses
	continuation if the *ambiguous* DC is chosen under *with*	the target continuation if either the
	*competitor* condition, otherwise literal feedback.	*ambiguous* or *unambiguous* DC is chosen.
	**2-3 fillers**: the target continuation matches **the incompatible DC only**	
	Contextual condition: randomly assigned per item; alternatives: no competitor only	
	Literal feedback: “Player 2" (correctly) guesses the target if the “incompatible” DC is chosen.	
2nd	**12 stimuli**: the target continuation matches **both the ambiguous and unambiguous DCs**	
half	Contextual condition: target-prediting/ competitor-predicting/neutral (counterbalanced)	
	**Feedback biasing the unambiguous DC**: “Player 2”	**Literal feedback**: “Player 2” guesses the
	guesses the competitor continuation whenever the	target continuation if either the *ambiguous*
	ambiguous DC is chosen.	or *unambiguous* DC is chosen.
	The guesses are **unblinded** and displayed.	The guesses are **unblinded** and displayed.
	**2-3 fillers**: the target continuation matches **the incompatible DC only**	
	Contextual condition: randomly assigned per item; alternatives: blinded	
	Literal feedback: the guesses are unblinded and displayed	

With the restriction that each participant does not see the same first argument more than once, the 62 stimuli with counterbalanced contextual and alternative conditions were divided into 60 lists of 31 items each, following the task structure described above. Each half of the task contained 12-13 active stimuli and 2-3 fillers, which were randomly shuffled for each participant within each half of the task. The rest of the procedure is similar to the setup of Pretest 3. The participants were given the same instructions, except that they were also informed that the alternative continuations would be blinded in the second half of the task.

### 4.3. Participants

Nine hundred native English speakers (age range: 19–85, mean age: 35, 536 females) were recruited on Prolific.ac, and were randomly assigned to the *with* and *without pragmatic exposure* groups. Six hundred and eighty eight of them reside in the U.K, 156 in the U.S. and the rest in Canada, Ireland, Australia or New Zealand. They did not take part in any of the pretests nor Experiment 1. They took an average of 21 min to finish the task and were awarded 1.8 GBP plus and average of 1 GBP bonus for their contribution. Workers who had more than 15 wrong answers were removed and replaced^1^.

### 4.4. Analysis

The experimental design of experiment 2 allows us to address several questions.

1. Is there an effect of contextual constraint on connective choice in the setting with blinded continuation alternatives? For this, we analyse the data from the second half of the second experiment.
2. Does the result from the pretest 3 replicate? We can test this based on the first half of the experiment.
3. Does the experimental task formulation play a major role in connective choice? For this, we will analyse the rate of unambiguous connectives inserted in the first vs. second half of experiment 2, and vs. experiment 1.
4. Finally, we can investigate the effect of pragmatic experience on connective choice: comparing the with pragmatic exposure vs. without pragmatic exposure settings from experiment 2 will allow us to quantify the effect of the language game experience, such as feedback, on communicative success.

For each of these questions, we will analyse different subsets of the data using linear mixed effects regression models in R, as described above. The full random effects structure is used whenever convergence is achieved. When a smaller random effects structure had to be chosen, this will be reported with the specific model. In all analyses, we only consider instances where the participant chose the ambiguous or the unambiguous connective. Cases where the incompatible DC was chosen are ignored in the analysis (this happened only in 3% of cases).

### 4.5. Results

#### 4.5.1. Effect of Context in Blinded Condition

Our first analysis tests for the main effect of interest: whether discourse connective choice is affected by whether the context is target-predicting or competitor-predicting, when the alternative continuations are not explicitly shown. According to the RSA hypothesis, a rational speaker should prefer the unambiguous connective more strongly in the competitor-predicting condition. The information that the speaker has in this setting is identical to the information available in experiment 1, but this time, the task formulation and instructions are comparable to pretest 3, and participants have already experienced the task with visible alternative continuations during the first part of the experiment. We thus here analyse the second half of the experiment, where the alternative continuation are blinded, and collapse across exposure type (with vs. without pragmatic exposure). Random slopes by participant had to be removed since they couldn't be effectively estimated by the model (their random effects correlation was 1.0). A binomial mixed effects analysis with connective choice as a response variable and context type as a predictor variable shows no significant effect of context type (target-predicting context: β = 0.050, *z* = 0.644, *p* > 0.05; neutral context: β = 0.061, *z* = 0.840, *p* > 0.05). We therefore also performed a Bayes Factor analysis with Bayesian multilevel models. The same settings as Experiment 1 were used, except that the number of iterations was increased (4 chains x iter = 10,000; warmup = 1,000; thin = 1; post-warmup = 36,000) due to increased data size, such that the Bayes Factor analysis could converge. In line with the glmer model, the Bayesian multilevel model also shows no effect of context type (target-biasing context: *t* = 0.03; 95% *CI* [−0.13, 0.19], neutral context: *t* = 0.04; 95% *CI* [−0.11, 0.19]). The Bayes Factor (*BF*_01_) comparing the reduced model without context as predictor (H0) over the model including context as predictor (H1) is 368, indicating very strong evidence for H0. The value of *BF*_01_ is thus about 10 times larger than the *BF*_01_ we obtained in Experiment 1. We think that this can be explained by the much larger number of observations in experiment 2 (10, 834 observations from 900 workers vs. 2, 741 observations from 225 workers).

The mean rate of unambiguous connectives is at 74% both in the target-predicting context condition and the competitor-predicting context condition. Again, we find a statistically significant effect of semantic gap on connective choice, and no effect for contextual gap (see Table 6). These findings are consistent with experiment 1, but inconsistent with highly rational connective choice. We note that the overall rate of unambiguous connectives in this experiment is substantially higher than in experiment 1; we will analyse the effects of experimental design in more detail in section 4.5.3.

**Table 6:** Regression coefficients of the logistic linear mixed effects model including the responses from the blinded conditions (second half) of Experiment 2.

**Variable**	**β**	**SE**	**z**	**p**
Intercept	0.731	0.715	1.023	0.306
Target-predicting context	0.050	0.078	0.644	0.520
Neutral context	0.061	0.073	0.840	0.401
Semantic gap	1.958	0.658	2.974	<0.003^**^
Contextual gap	−0.978	0.738	−1.325	0.185

** *p < 0.01*.

#### 4.5.2. Replication of Pretest 3

We next analyse the data from the first half of the experiment. The setup here is identical to pretest 3, except that all three different contexts are included, not just the neutral context. We do however not expect any difference between the context conditions, as the possible alternative interpretations of the hearer are shown explicitly. If the contextually predicted alternative is not presented among the alternative continuations, we do not expect this alternative to affect connective choice. However, we do expect to replicate the effect of competitor presence among the explicitly shown alternatives on connective choice.

A binomial linear mixed effects model (see also Table 7) showed a statistically significant effect of competitor presence among the explicitly shown alternatives (β = 0.446, *z* = 5.974, *p* <0.001), in line with pretest 3. As expected, we do not find a significant effect of either context condition (β = 0.001, *z* = 0.010, *p* > 0.05) for the target-predicting context compared to the competitor-predicting context, and (β = 0.061, *z* = −0.940, *p* > 0.05) for the neutral context compared to the competitor-predicting context when alternative completions are shown.

**Table 7:** Regression coefficients of the logistic linear mixed effects model including the responses from the first half of Experiment 2.

**Variable**	**β**	**SE**	**z**	**p**
Intercept	0.904	0.143	6.303	<0.001^***^
With competitor	0.446	0.075	5.974	<0.001^***^
Target-predicting context	0.001	0.068	0.010	0.992
Neutral context	−0.061	0.065	−0.940	0.347

*** *p < 0.001*.

#### 4.5.3. Comparison Across Experimental Designs

Table 8 provides an overview of the proportion of instances where participants chose an *unambiguous* DC instead of an *ambiguous* DC across the different experimental designs in this study. (Cases were participants selected an incompatible DC are not counted in the table.)

**Table 8:** Mean unambiguous DC proportion per participant under various conditions in Experiment 1 and 2.

	**Exp. 2: with pragmatic exposure**			**Exp. 2: without pragm. exp**.		**Exp. 1**
	**No**	**With**	**Blinded**	**No**	**Blinded**	**Unknown**
	**competitor**	**competitor**	**(second half)**	**competitor**	**(second half)**	
	**(first half)**	**(first half)**		**(first half)**		
Overall	70%	77%	75%	70%	72%	64%
Target						
-predicting	71%	78%	75%	70%	72%	65%
Competitor						
-predicting	71%	77%	75%	68%	73%	65%
Neutral	69%	76%	74%	71%	72%	63%

We ran a binomial linear mixed effects model with connective type as the response variable and experimental design (with vs. no competitor vs. expt1 vs. blinded with pragmatic exposure vs. blinded without pragmatic exposure) as the predictor variable; the blinded without pragmatic exposure condition was used as the baseline condition, to test whether results are significantly different from the pretest 3 setting or the setting from experiment 1.

First, we found that there are significantly more insertions of unambiguous connectives in the blinded condition with pragmatic exposure, compared to no pragmatic exposure (β = 0.178, *z* = 2.798, *p* < 0.01). This means that experience with ambiguity in the first half of the experiment does affect participants' connective choices, such that they are more likely to choose unambiguous connectives subsequently. It is possible that this effect is the result of *learning* from unsuccessful communication during the experiment (i.e., where the comprehender chose a competitor completion)

As expected, there is an even stronger effect for the with-competitor condition, where the competitor interpretations are shown explicitly (β = 0.226, *z* = 2.789, *p* < 0.01), compared to the blinded no pragmatic exposure baseline.

We also find a graded effect in the other direction: there are significantly fewer insertions of unambiguous connectives in the no-competitor condition, where the alternatives are explicitly limited to non-confusable options (β = −0.180, *z* = −3.063, *p* < 0.01, see also Table 9); people choose unambiguous connectives less often when they know that the alternatives don't include any instances which would lead to misunderstandings.

**Table 9:** Regression coefficients of the logistic linear mixed effects model including the responses from Experiment 1 as well as both halves of Experiment 2.

**Variable**	**β**	**SE**	**z**	**p**
Intercept	1.212	0.155	7.834	<0.001^***^
Expt-1	−0.472	0.094	−5.030	<0.001^***^
Blinded (with prag. exposure)	0.178	0.064	2.798	<0.01^**^
No competitor	−0.180	0.059	−3.063	<0.01^**^
With competitor	0.226	0.081	2.789	<0.01^**^

*The base level of the predictor variable is the blinded condition of the without pragmatic exposure version of Experiment 2*.

** *p < 0.01,*

*** *p < 0.001*.

Furthermore, we also see that there is an even lower rate of unambiguous connectives in the experiment 1 design (β = −0.472, *z* = −5.030, *p* < 0.001, compared to the blinded condition). This indicates that, even though the same information is available to the speaker in both cases, there is an influence of experimental task: participants are more aware of the existence of interpretation alternatives on the side of the hearer in the blinded setting, and therefore are also aware of the risk of misunderstanding, which leads them to prefer the unambiguous connective.

These results hence reveal a graded effect of restriction on alternative interpretations: when the possible interpretations are not limited, the speaker will use more precise DCs than in situations where the alternatives are limited to non-confusable relations; the speaker is sure that the listener won't misunderstand. When a confusable interpretation is explicitly included in alternatives, the speaker will, in turn, be more aware that a misunderstanding is possible, compared to when interpretations aren't explicitly provided. As the difference between the with and without pragmatic exposure conditions shows, participants' previous experience in the gamified task affects their choice. They are more aware of the chance of mis-interpretation if they have previously seen confusable alternatives in the “training phase,” or even received some corrective feedback.

### 4.6. Summary and Discussion

Summarizing the results of Experiment 2, we found that contextual expectation of a competitor discourse relation does not have the same effect as presenting it explicitly as a possible continuation. These findings replicate the results of experiment 1. We therefore conclude that the lack of effect in experiment 1 cannot be attributed to the instructions of the task, but rather to the not explicitly listing the alternative listener interpretation options. It is possible that it is too difficult for the speaker to reason about the discourse expectations that the context raises for the listener.

The empirical results we found here are thus not consistent with our expectations based on the rational speech act model: while we had expected to find an effect of discourse relation expectation on connective choice, similar to the effect found in pretest 3, we were not able to detect any such effect, and in fact, our Bayesian Factor analysis indicates that the data strongly support the null hypothesis.

The argument we made here is based on a qualitative prediction of the RSA theory, and qualitative results from our empirical data. As the RSA framework is capable of making quantitative probabilistic predictions, it would be possible to also test more exact quantitative predictions. The required ingredients include the prior distribution of the salience of a relation based on the biasing contexts, which serves as the literal listener model (Equation 3) and a function that defines the production cost of a given DC (Equation 2). Both measures could be obtained empirically in separate experiments.

We assume that the production costs do not vary across experimental conditions, and the main driving factor of the effect would be the discourse relation inferences of the listener after having perceived the connective. Based on our prestest 2, we believe that our experimental manipulation was effective in changing comprehender interpretations, and that there would thus be a substantial difference between context conditions also in an experiment that collects this prior probability more directly. However, given the lack of even a qualitative effect in our data, even when we used a very large number of participants in experiment 2, we think that it is not very promising to proceed to a more quantitative comparison at this point.

The comparison of experimental designs provided evidence that gamified elements such as the explicit listing of alternatives, and experience with the task induce participants to choose an unambiguous connective more often. These results thus indicate that gamification of the task affects rational reasoning and thereby the results of the RSA study.

## 5. Overall Discussion and Conclusion

The RSA model states that speakers reason about the interpretation of the listener and weigh the cost of an utterance against its utility in avoiding misunderstandings. According to this theory, it is predicted that when the listener is likely to confuse an intended discourse relation with another relation, the speaker should avoid the (albeit temporary) misunderstanding by using a more informative utterance, by using a DC that signals the target relation more exclusively. Following the success of predicting human behavior in a variety of language processing tasks, such as the production of referring expressions, the RSA account had also been shown to make correct qualitative predictions on the speaker's choice of DCs in an language game experiment by Yung and Demberg (2018). Language games of this kind are widely used to explore pragmatic inferences in contexts because they allow precise manipulation by explicitly displaying the a set of listener interpretations to the speaker.

The current study set out to test RSA's prediction on discourse relation production using a methodology of improved ecological validity by removing the explicit statement of what the interpretations of the listener might be. Instead, manipulation on the preceding context is used to elicit discourse expectations, which either match or do not match with the target discourse relation to be conveyed by the speaker. We hypothesized that a situation where a confusable discourse relation is highly expected in context will lead to similar increased demand in choosing a suitable connective to avoid temporary misinterpretations by the listener.

Experimental results show that the context manipulation is successful in that the connective marking the target relation is inconsistent with the expected relation and can hence help to correct listener expectations early on.

However, our experiment 1, which did not explicitly show the discourse expectations, reveals that, contrary to our hypothesis, the preference to produce a particular discourse relation with a specific, unambiguous DC does not depend on whether the target relation or other competing relations are expected. Further experiments, using a modified language game design, confirms that the contextual expectation of a competitor discourse relation does not affect the production of the DC.

We however did find that participants use more informative utterances when the listener's interpretation is unrestricted than when the interpretations are restricted to non-confusable alternatives. That is, when the listener can see that there is no risk of misinterpretation, they do not use unambiguous connectives as much, but also use ambiguous ones (which in this context, in fact are also unambiguous). These results indicate that people do reason about the listener's interpretation, consistent with earlier findings, but only when the interpretation alternatives are easily accessible. Our results are consistent with an account according to which speakers adopt general strategies, instead of reasoning about each case, in line with earlier results by Vogels et al. (2020). One such strategy that we observed here was to more often choose unambiguous connectives, if ambiguity had been experienced earlier in the study.

How can the absence of any effect of contextual expectation be explained? We see two possible options:

- inferring discourse expectations and reasoning about them is too difficult, therefore participants don't do it (i.e., they only engage in reasoning when the discourse expectation inference step is done for them by the experimental design).
- they do infer discourse expectations and reason about listener interpretations, but feel that it's not necessary to disambiguate the relation as the content of the second argument of the relation will eventually lead to full disambiguation anyway.

Regarding option (a), let's first take a step back: the RSA crucially states that speakers reason about the interpretation of the listeners in order to maximize the informativeness of the utterance. An underlying assumption is that they are equipped with the necessary resources, such as computational resources and background knowledge, to do so. Communication of concrete meanings, such as reference to particular objects or numerical quantities, have been extensively studied in existing work. Oftentimes, the alternative interpretations from the point of view of the listener were also directly available to the speaker in those studies, consider for instance referring expression generation. In the main experiment, the subjects had to do another level of inference: to rationally select an informative connective, they would have to reason about what the listener would expect. These discourse expectations are not only abstract concepts (which may be more difficult to juggle in memory), but they also are not present in the visible context of the interaction.

The results from Yung and Demberg (2018) and pretest 3 demonstrate that speakers *can* choose connectives in order to avoid misinterpretations on the side of the listener, pretest 2 further demonstrated that the stimuli do give rise to expectations, and earlier work has provided ample evidence that listeners generate discourse expectations during comprehension (Sanders and Noordman, 2000; Rohde et al., 2011; Canestrelli et al., 2013; Rohde and Horton, 2014; Xiang and Kuperberg, 2015; Scholman et al., 2017; Van Bergen and Bosker, 2018; Schwab and Liu, 2020; Köhne-Fuetterer et al., 2021). However, there is no direct evidence that speakers also simulate the discourse expectations that listeners would generate.

The RSA theory does not provide explicit limits or definitions as to when a speaker reasons about a listener, and for what linguistic phenomena or under which situational circumstances this reasoning would be too effortful. In fact, a common criticism of RSA (and Gricean pragmatics) is that it falls short in explaining speaker productions: utterances are sometimes longer than they need to be, underinformative or ambiguous (Engelhardt et al., 2006; Gatt et al., 2013; Baumann et al., 2014; McMahan and Stone, 2015), and speakers also sometimes fail to take listener perspective into account when generating referring expressions (Horton and Keysar, 1996; Lane and Ferreira, 2008; Yoon et al., 2012).

These findings have lead to discussions as to whether speakers really always behave rationally, and more specifically, whether speakers reason about listeners in all cases, and how many levels of recursion in reasoning should be considered (Degen and Franke, 2012; Franke and Degen, 2016) (in most previous models, the default is set to 2 levels of recursion). Yuan et al. (2018) explored these questions in the context of reference games and found that pragmatic listeners and speakers always outperform their literal counterparts and that model performance becomes more accurate as more levels of recursion are assumed.

Yuan et al. (2018) also explored the effect of limiting the number of considered alternatives, and found that this does not detrimentally affect model results (in fact, it improves model fit). Note that the results found in the present experiment do not require a large number of alternatives: strictly speaking, even reasoning about the top-1 alternative from the point of view of the listener would be sufficient to elicit an effect of context on discourse connective choice. Also, the number of levels of recursion depth required in the reasoning in our experiment is not large—default 2-level recursion would be sufficient. Therefore, those prior concerns do not explain why we fail to find an effect here.

In summary, while it is well-known that there can be differences between individuals as to how deeply they engage in the reasoning process, there has previously been little discussion with respect to the potential differences in cognitive difficulty of making a single reasoning step. Our study hence sheds light on a potential additional source of limitation with respect to reasoning about the interlocutor, outside of recursion depth of the number of alternatives that need to be considered.

Option (b) is a possible criticism for the design in experiment 1, in particular if dropping the assumption of *incremental* RSA. However, if this was the determining factor of why there was no difference between the context conditions, we should have seen an effect of context in the blinded conditions in the second part of experiment 2: in this setting, people were very aware that the task of their listener was to guess the second argument, so not providing a helpful hint for that guess would be pretty non-cooperative. Therefore, the outcome of experiment 2 doesn't seem compatible with this explanation.

In terms of methodological contribution, we found that the widely used gamified experimental design substantially affects results, even in a setting where the experimental items are the same. We designed an experimental structure that tests the speaker's DC production in relation to different levels of guidance of pragmatic reasoning. Minimum guidance was provided in Experiment 1: the speaker did not know if the communication was successful or not, as no feedback was given. Maximum guidance is provided in the previous work by Yung and Demberg (2018) and the first half of the *with pragmatic exposure* version of Experiment 2: the speaker learns that the listener might guess the competitor relation if the utterance is not specific enough. An interesting condition between these two extremes was the blinded condition in the second half of experiment 2. Here, the task is identical to the maximum guidance setting, but the knowledge available to the speaker is identical to the minimum guidance setting. Here, we see a generic increase in the use of unambiguous connectives compared to the minimal guidance setting, but no condition-specific increase as would have been expected according to the RSA.

We therefore conclude that gamification of the task, which encourages reasoning about alternatives, boosts RSA-consistent behavior. However, it remains unclear to what extent the findings from a language game actually represent people's normal language production. One direction for future work is thus to validate RSA-styled production and interpretation outside the assumption of a toy world for other phenomena.

We think that alternative experimental designs should be explored, which seek for free production of utterances given a manipulated prompt or situation, such as a story generation task given a sequence of pictures. While such a free production task is much closer to naturalistic language use, it is not trivial to elicit specific discourse relations and closely control the experimental conditions in such a design. However, given enough data, it is still possible to collect a distribution of intended discourse relations and the corresponding connectives. Crowd-sourcing could be an effective way to collect such a database, as the additional noise introduced through the less controlled experimental design might be counter-weighed by a larger number of participants.

Furthermore, the results of this experiment lead us to the question of what it takes for the speaker to engage in reasoning about the listener interpretation. When do speakers consider listener misinterpretation risk, and under which circumstances is this calculation too effortful? One possibility might be that reasoning of the speaker about the listener might be triggered only when a small set of explicit alternatives is available and in full view, such that it doesn't need to be held in memory. This question thus addresses the generality with which the RSA account holds: in principle, it is formulated such that it covers reasoning about listener interpretations independent of the form in which they are available to the speaker. However, experiments conducted so far have only addressed a small area of the possible production phenomena that RSA could be applied to, and have addressed these questions in settings where the alternative listener interpretations are in a shared visible space.

## Data Availability Statement

The datasets presented in this study can be found in online repositories. The names of the repository/repositories and accession number(s) can be found at: https://osf.io/q32vj/?view_only=61f5a72585c74690ac2bbc6222403c76.

## Ethics Statement

The studies involving human participants were approved by Deutsche Gesellschaft für Sprache (DGfS). The participants provided their written informed consent to participate in this study.

## Author Contributions

FY and VD conceived the idea, designed the experiments, and wrote the manuscript. FY carried out the experiments and analyzed the data. All authors contributed to the article and approved the submitted version.

## Conflict of Interest

The authors declare that the research was conducted in the absence of any commercial or financial relationships that could be construed as a potential conflict of interest.

## Footnotes

^1^A wrong answer refers to a DC that does not match the target continuation, so both the ambiguous and unambiguous DCs are considered correct, even in cases where it results in a wrong guess.
